# Termite inspired algorithm for traffic engineering in hybrid software defined networks

**DOI:** 10.7717/peerj-cs.283

**Published:** 2020-08-17

**Authors:** R Ananthalakshmi Ammal, Sajimon PC, Vinodchandra SS

**Affiliations:** 1 Cyber Security Group, Centre for Development of Advanced Computing (CDAC), Thiruvananthapuram, Kerala, India; 2 Computer Centre, University of Kerala, Thiruvananthapuram, Kerala, India

**Keywords:** Multi Commodity Flow (MCF), Software Defined Networking (SDN), Termite-inspired, Traffic Engineering, Hybrid SDN

## Abstract

In the era of Internet of Things and 5G networks, handling real time network traffic with the required Quality of Services and optimal utilization of network resources is a challenging task. Traffic Engineering provides mechanisms to guide network traffic to improve utilization of network resources and meet requirements of the network Quality of Service (QoS). Traditional networks use IP based and Multi-Protocol Label Switching (MPLS) based Traffic Engineering mechanisms. Software Defined Networking (SDN) have characteristics useful for solving traffic scheduling and management. Currently the traditional networks are not going to be replaced fully by SDN enabled resources and hence traffic engineering solutions for Hybrid IP/SDN setups have to be explored. In this paper we propose a new Termite Inspired Optimization algorithm for dynamic path allocation and better utilization of network links using hybrid SDN setup. The proposed bioinspired algorithm based on Termite behaviour implemented in the SDN Controller supports elastic bandwidth demands from applications, by avoiding congestion, handling traffic priority and link availability. Testing in both simulated and physical test bed demonstrate the performance of the algorithm with the support of SDN. In cases of link failures, the algorithm in the SDN Controller performs failure recovery gracefully. The algorithm also performs very well in congestion avoidance. The SDN based algorithm can be implemented in the existing traditional WAN as a hybrid setup and is a less complex, better alternative to the traditional MPLS Traffic Engineering setup.

## Introduction

In today’s world, the dependency on the Internet is huge with everything becoming cloud-centric. Digital transformation is happening in enterprises, government departments and organizations worldwide, and handling real time network traffic with the required Quality of Services and optimal network resource utilization is very challenging. Many of the service providers and enterprises make use of redundant links for meeting the increased bandwidth requirements and backup paths. In the existing traditional networks, for routing the traffic flow among the multiple available network links, many static and dynamic algorithms exist (Fang & Wang, 2009; Lee et al., 2002; Lahoud, Texier & Toutain, 2005). There is a strong motivation to efficiently utilize the available links based on user priorities and dynamic bandwidth demands with minimal human intervention. In most cases, costly Multi Protocol Label Switch (MPLS) links are used and the bandwidth allocation is configured in a static manner. The links are overprovisioned and it has been observed that the utilization of the links is of the order of 40–50% only, with the highest bandwidth link carrying the bulk of traffic and the remaining links serving as backup paths. Typically, Equal Cost Multi Path routing (ECMP) (Hopps, 2000) is used to assign the flow among the existing links. Service differentiation is achieved through prioritization and by setting the Differentiated Services Code Point (DSCP) bits in the IP header. Many of the methods do not take into consideration the current state of the network including link availability and congestion status. Traffic Engineering is used to control the network resources efficiently and to optimize the performance of the network. Traditionally, the Link State Routing Protocol, Open Shortest Path First (OSPF) (Moy, 1998) in IP networks and Multi-Protocol Label Switching–Traffic Engineering (MPLS_TE) (Srinivasan, 2004) in MPLS networks, are some of the technologies used for network traffic engineering. But these technologies have several drawbacks, including their complexity in configuration and great performance overhead. Another major constraint is the lack of trained manpower to setup and maintain the complex router configurations for MPLS-TE. Equal Cost Multi Pathing (ECMP) (Lappeteläinen, 2011) technology cannot split traffic across two links with unequal weights. Moreover, in traditional networks, the control and data forwarding are tightly coupled in the distributed routers and switches, which makes traffic engineering difficult.

Software Defined Networking (SDN) (McKeown et al., 2008) is a network paradigm which is currently being adopted, where the data plane and control plane are separated and there is a logically centralized Controller having a view of the entire network. This centralized architecture enables to overcome many routing issues and flow paths can be assigned dynamically based on network constraints. SDN provides exciting prospects for Network Traffic Engineering and Optimization. With the separation of data plane and control plane, as well as with the support for network programmability, new SDN based traffic management can be explored. The centralized Controller collects the network information including the network topology, current link status, link utilization, application requirements such as QoS and priority requirements. With this information, the SDN Controller should be able to support traffic load balancing, QoS guarantee and traffic management. The openness of the SDN Controller is an added advantage which supports the network programmability to optimize the allocation of network resources. Many of the current SDN implementations are based on the OpenFlow protocol and as per the architecture of the protocol, every first packet of the flow arriving at the data forwarding device causes a message to be initiated to the Controller seeking forwarding rules, to process the flow. This helps the management of traffic flow to be more flexible and dynamic.

Even though SDN promises many exciting opportunities to ease the operation and management of networks, traditional networks setup with the vendor specific devices cannot be replaced by SDN in the near future. Moreover, no changes can be made to these traditional forwarding elements such as routers and switches, which are mostly proprietary resources. So, traffic engineering solutions have to cater to hybrid network models where the traditional network and SDN can coexist. Many Hybrid SDN models have been described (Vissicchio, Vanbever & Bonaventure, 2014), which support for the effective communication between the SDN Controller and other traditional network resources. In this paper, traffic engineering solution for a hybrid SDN model where the SDN Controller is integrated with the traditional network, with minimal SDN enabled devices is proposed. The objective of the paper is to develop a solution for Wide Area Network traffic engineering in a Hybrid SDN model, that can effectively manage the dynamic bandwidth demands, avoid congestion and achieve better link utilization with the required prioritization.

Traditional link state or distance vector shortest path algorithms perform very well in static networks and fail in dynamic conditions with unstable topology and traffic conditions. To handle the dynamic conditions in a real network scenario, bio-inspired algorithms have been proposed, as biological systems are resilient to failures, adaptive to changes in external environment and scale well with their distributed nature. Many bio-inspired algorithms based on swarm intelligence capabilities of ants, bees, and bird flocks are being used in real world scenarios effectively. The published bio-inspired algorithms are applicable in traditional networking and only a very few are available for SDN. We propose a termite inspired optimisation algorithm to be executed in the SDN Controller, based on the sophisticated swarm cognition of termites, the blind and cryptic eusocial insect.

Our major contributions in this paper include:

• First, a practical solution for the existing Wide Area Network (WAN) infrastructures to enhance the performance of traffic management by introducing an SDN Controller at the network gateway. The system design helps in incremental deployment of SDN in traditional networks for achieving traffic engineering with SDN support. We used the existing standard protocols to build our architecture.
• Second, a new termite inspired optimization algorithm for traffic engineering is deployed in the SDN Controller with the objective of achieving maximum flow over the path dynamically, subject to link capacity, congestion, priority of traffic and link availability. The algorithm shall be capable of meeting the elastic bandwidth demands with support for better link utilization, congestion avoidance, traffic prioritization and handling of link failures
• Third, several experiments were conducted to evaluate the performance of the newly proposed Termite inspired algorithm and it is seen that the proposed hybrid design with the algorithm executed in the SDN Controller can very well replace the complex and costly MPLS -TE configurations.

## Related Works

In Traffic Engineering, the requirements of multiple network users have to be effectively managed by avoiding congestion of paths and better utilization of the available paths. In order to achieve the same, the topology of the network, state and utilization of all network paths and overall network load have to be collected. In most of the IP based traditional networks, the highest bandwidth shortest path is selected as the best path and other path serves as the backup path. Whenever dynamic traffic routing is to be carried out, many problems such as routing instability and packet reordering delays crop up.

SDN offers a wide spectrum of possibilities to manage the traffic in a network. The logically centralized Controller in SDN has a global view of the network and its status. Hence the SDN Controller can perform Traffic Engineering (TE) in a more flexible and effective manner. In contrast to traditional networks, the entire data layer traffic is controlled by the SDN Controller. This helps to plan the traffic forwarding strategy much better, taking into consideration the optional link utilization and flow characteristics. There are existing works such as Microsoft SWAN (Wattenhofer et al., 2016), Google B4 (Jain, Kumar & Mandal, 2013) and BwE (Kumar et al., 2015) which focus on the WAN optimization problems in SDN based WAN. They are SDN based WANs implemented for enhancing the network performance and utilization through better traffic scheduling. The Bandwidth Enforcer Algorithm allocates bandwidth to competing applications based on flexible policy configured by bandwidth functions and claims to achieve better bandwidth utilization. In the above cases, the equivalence of ECMP is used for link load balancing. This created problems for elephant flows, where all the packets belonging to a flow were forwarded along the same path, causing load imbalance. To support dynamic management of elephant flows, other SDN based traffic management systems such as Hedera (Al-fares, 2010), Mahout (Curtis, Kim & Yalagandula, 2011) and MicroTE (Benson et al., 2011) were developed. DevoFlow (Curtis et al., 2011) and DIFANE (Yu et al., 2012) are two OpenFlow protocol based traffic load balancing mechanisms.

The above traffic management mechanisms are ideal and functional in SDN only environments. The traditional network resources are not going to be replaced by pure SDN compliant resources in the near future. So, the solution has to cater to hybrid environment with traditional network and SDN. Agarwal, Kodialam & Lakshman (2013) have proposed a traffic engineering model with minimal SDN enabled forwarding devices. The solution mentioned does not take into consideration the QoS requirements of real time network traffic where there are instant messages or voice traffic, as well as the link availability and congestion status. He & Song (2015) have proposed traffic engineering algorithm considering the SDN only traffic, traditional devices traffic and hybrid mode traffic. The number of SDN devices deployed has an impact on the performance and theoretical approximations are provided for the traffic engineering problem. Guo et al. (2014) have published a metaheuristic SOTE algorithm for traffic optimization in hybrid SDN/OSPF network. The performance is achieved only when 30% of the devices in the network are SDN compliant.

There are a few research activities related to application of bio-inspired algorithms to SDN. An Ant Colony Optimization (ACO) approach to flow routing in SDN environments have been published by Dobrijevic, Santl & Matijasevic (2015). An ACO-based heuristic algorithm was used to calculate Quality of Experience (QoE) -aware paths that conform to traffic demands. QoE aware shortest paths were found in a low running time. A link load balancing algorithm based on Ant Colony Optimization (LLBACO) was also published by Wang et al. (2017), which used the search rule of ACO and took link load, delay and packet-loss as impact factors. For maintaining link load-balancing and reducing end-to-end transmission delay, the widest and shortest path in the all paths were calculated. Particle Swarm Optimization based algorithm to maximize the overall flow with support for QoS is published by Yao et al. (2016). These bio-inspired algorithms have not taken into consideration the different constraints including the case of link failures and do not switch over to alternate paths when there is a link failure.

## The Hybrid SDN Model

The problem of handling the traffic across the existing wide area links, with limited capacity, prioritized applications and link failure conditions is a very common one. Replacing all the existing WAN routers with MPLS-TE supported routers is not practical. The problem scenario is in one of the organizations, where there are three WAN links with limited capacity connecting different branches of the organization. There are different classes of end user applications, with varying priority. An optimal solution for the usage of the existing resources with limited bandwidth, avoiding congestion and graceful recovery from link failure is the need of the hour. An SDN based solution with Traffic Engineering support is deployed as a cost effective one.

**Figure 1:**
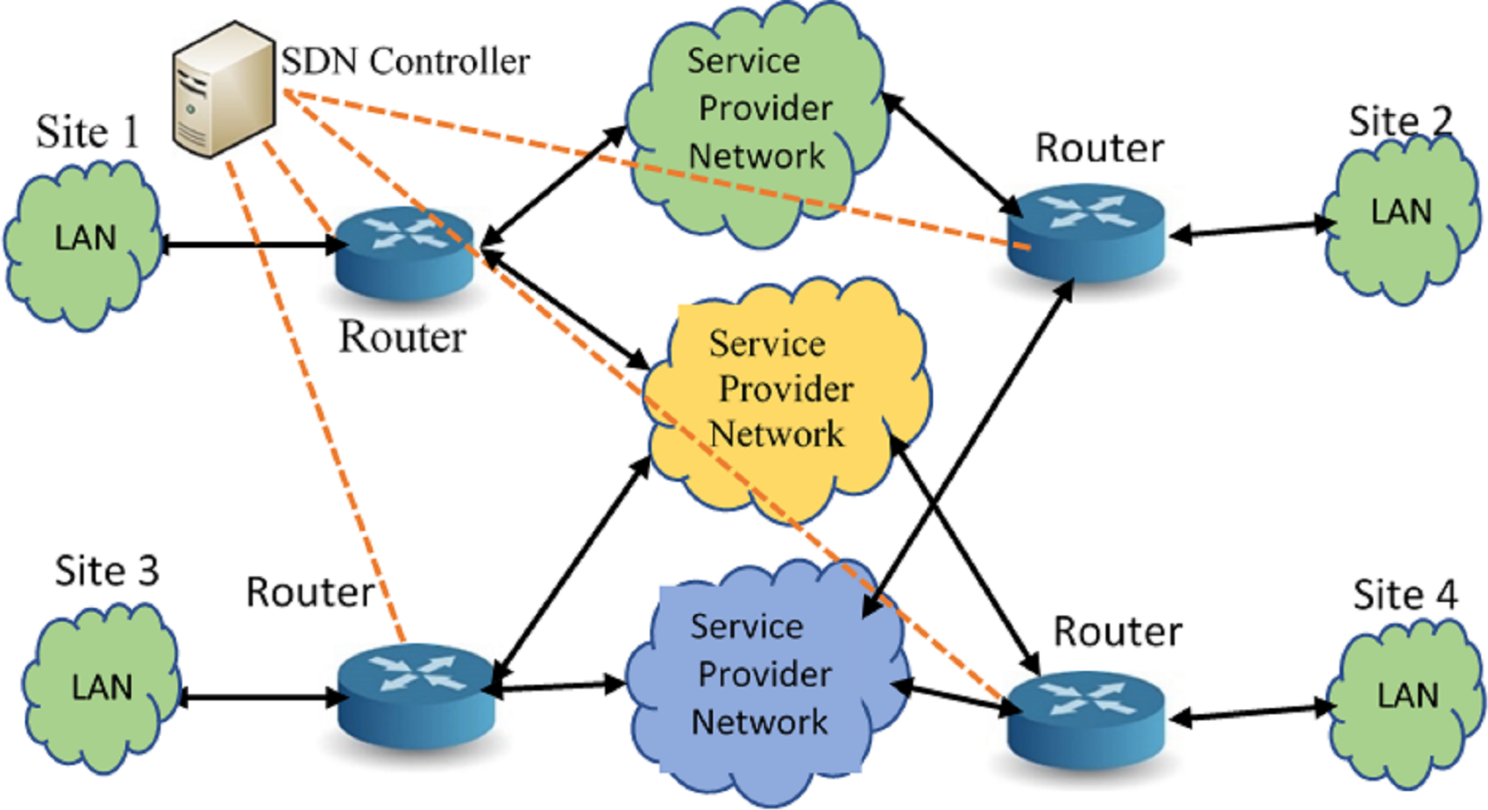
A sample logical model of the hybrild SDN setup.

In the proposed system model, SDN Controller is introduced into the network and is connected to the gateway router. The SDN Controller runs all the applications such as identifying the topology of the network, monitoring the network availability, bandwidth utilization and computes the forwarding paths dynamically. The SDN Controller is connected to the gateway router using an OpenFlow enabled switch, which acts as merely a data plane device for the SDN Controller. The gateway router uses the Link state or Distance Vector routing protocols to forward the packets through the WAN links. The SDN Controller is capable of gathering traffic information, executes the traffic optimization algorithms and disseminates the traffic flow paths to the gateway router, shaping the traffic. SDN Controller thus provides the necessary ecosystem for deploying the traffic optimization solution. Figure 1 shows the mesh network connecting different sites through WAN links from multiple service providers.

### Traffic optimization problem

Any network traffic engineering system will be having an optimization system which optimizes the network resource utilization, avoids network congestion and controls the network traffic. The complexity of the optimization algorithm increases as the number of constraints increase. Given multiple links, the problem of routing and congestion control can be considered as a category of the general Multi-Commodity Flow (MCF) problem. The maximum throughput MCF is a classical optimization problem that handles issues of congestion and bandwidth management in connection-oriented network architectures, with the objective to maximize the total flow that can be routed, subject to edge-capacities. Many algorithms based on linear programming are available (Castro & Nabona, 1996; Hall, Hippler & Skutella, 2007) for Maximum MCF with minimum cost problem. These algorithms are based on static flow and do not take into consideration the real scenarios such as link unavailability, elastic bandwidth demands, traffic prioritization etc. This optimization problem is considered as an NP-Complete problem (Johnson, Lenstra & Kan, 1978). In general, for NP-complete problems, there exists no polynomial deterministic algorithm that can solve a real-world problem in a reasonable amount of time. So, in order to solve the real network scenario, metaheuristic approaches have been considered. In this paper we present a bio-inspired algorithm based on the termite behavior model applied to traffic management. The proposed termite inspired optimization algorithm executed in the SDN Controller helps to achieve better link utilization, required prioritization, dynamic bandwidth demands and congestion avoidance.

### Behaviour of termites

Termites are eusocial insects which are blind and cryptic in nature and live in colonies comprising of nearly one million termites depending on the species. The eusocial insects exhibit complex behavior like group decision making and nest construction. The mechanism of sensible collective decision making, also known as swarm intelligence, is now being applied in many optimization problems. Among the eusocial insects such as ants, bees and termites, termites have a sophisticated swarm cognition (Turner, 2011), in which, apart from the interaction among the members of the swarm, they also interact with the external environment. The termite colonies exhibit division of labor and consist of categories with a specific function in the community. There are three principal categories namely –the workers, the soldiers and the reproductive. Termites communicate through vibrations and chemical signals (pheromones) as they are blind. Pheromones support the termite social structure. They recognize their nest mates by scent. Each colony develops its own scent. Termites also secrete pheromones to mark the trail to food or alert the colony to danger. The foraging termites lay down a trail of pheromone, which they secrete from glands on their abdomen, while tunneling underground. The intensity of this type of pheromone is increased when a food source is located. This helps to recruit more workers to the food source. Similarly, whenever there is an attack or invasion to the colony, the soldiers secrete another type of pheromone, whose scent gives signal to other termites of the impending danger.

Environmental factors also affect the termite colony and their behaviour. The two major factors are the temperature and moisture. Foraging activities depend on soil temperatures and is very minimal during extreme cold and hot temperatures, depending on the termite inhabiting regions. Depending on the species, optimal temperatures range from 21 °C to 36 °C. Termites depend on moisture either from environment or from food source. Construction of tubes and tunnels by termites, which exhibit their engineering skills, help in preserving the right amount of moisture internally, in turn preserving their soft cuticle. The stochastic decision-making process of the termites in a colony decide their moving pattern. A termite selects its moving pattern based on the local pheromone intensity. Thus, each termite is able to perform complex tasks using simple rules of behavior. The termite swarm adaptively responds to the newly encountered conditions. This model can be applied to many dynamic optimization problems. This intelligent behavior of termites has been instrumental in proposing a novel optimization method for WAN Traffic management. The proposed bioinspired algorithm based on Termite behaviour can be used to solve the constrained multi commodity max flow problem

### Maximum multi-commodity flow problem

A Wide Area Network can be considered as a directed graph G (V, E) where V is the set of Edge Routers and E is the set of WAN links. Between two WAN routers, multiple links coexist. The objective is to maximize the traffic flows through the WAN links subject to link availability, capacity, traffic demand, congestion and priority. Routing the flows through a single path or multiple paths can be considered as a Multicommodity Flow Problem, where the paths are already identified. It is well known that there are no polynomial time algorithms to compute the Multicommodity Maximum Flow for multiple unicast commodities subject to constraints (Hall, Hippler & Skutella, 2007). Let k denote the commodity and ${f}_{\mathrm{i,j}}^{\mathrm{k}}$ the flow of the commodity k through the edge (i,j) ∈ E, then the total flow of the commodity k from the source *s* to the sink *t* for the directed graph, can be depicted as follows

${\sum }_{\mathbi{j}}{\mathbi{f}}_{\mathbi{i},\mathbi{j}}^{\mathbi{k}}-{\sum }_{\mathbi{j}}{\mathbi{f}}_{\mathbi{j},\mathbi{i}}^{\mathbi{k}}={\mathbf{F}}^{\mathbi{k}}$ where i=s_k_

${\sum }_{\mathbi{j}}{\mathbi{f}}_{\mathbi{i},\mathbi{j}}^{\mathbi{k}}-{\sum }_{\mathbi{j}}{\mathbi{f}}_{\mathbi{j},\mathbi{i}}^{\mathbi{k}}=0$ where i≠s_k_,t_k_,

${\sum }_{\mathbi{j}}{\mathbi{f}}_{\mathbi{i},\mathbi{j}}^{\mathbi{k}}-{\sum }_{\mathbi{j}}{\mathbi{f}}_{\mathbi{j},\mathbi{i}}^{\mathbi{k}}=-{\mathbf{F}}^{\mathbi{k}}$ where i=t _k_.

The objective function is to maximize the total flow through the links: ***Max***∑_***k***_|**F**_***k***_| (1)

Considering the capacity of the edge as c_i,j_ , the flow is subject to $0\leq \left\vert {\mathbi{f}}_{\mathbi{i},\mathbi{j}}^{\mathbi{k}} \right\vert \leq {\mathbi{c}}_{\mathbi{i},\mathbi{j}}^{\mathbi{k}}$ for the kth flow and $0\leq {\sum }_{\mathbi{k}}{|}{\mathbi{f}}_{\mathbi{i},\mathbi{j}}^{\mathbi{k}}{|}\leq {\mathbi{c}}_{\mathbi{i},\mathbi{j}}$ for all the flows along the edge. (2)

The edge availability of the arc (i,j) is {0,1} ∀ (i,j) ∈ E (3)

For a link flow *f*i,j, the congestion factor is $\beta = \frac{{\sum }_{\mathbi{k}}{|}{\mathbi{f}}_{\mathbi{i},\mathbi{j}}^{\mathbi{k}}{|}}{{\mathbf{c}}_{\mathbf{i},\mathbf{j}}}  $ and

for congestion avoidance 0 ≤*β* <1 $, \forall \left( \mathbf{i},\mathbf{j} \right) \in \mathbf{E}$ (4)

The traffic demands are varying, with different levels of priorities. The priorities can be assigned based on predefined policies.

### Bio-inspired algorithm based on termite behaviour

The bioinspired algorithm is based on termite colony behavior, where there are different categories of termites existing in a colony. The termite colony can be modeled as a flow commodity to be transported from source to sink. It is assumed that all the flows belonging to a commodity are transported through one link from source to sink to avoid latencies and reordering complications at the sink. This is modelled from the behavior that the termites belonging to a colony identify one another based on the chemical scent generated from their bodies. Each worker termite of the termite colony can be considered as part of the flow to be transmitted from source to sink and they follow the trail based on the pheromone value. It is assumed that the multiple paths from source to sink are already known to the Controller identified as part of the network topology discovery.

### Pheromone updates

The movement of termites is controlled by pheromone content on the path and analogous to the same, the SDN Controller maintains the Pheromone details of the various paths of the discovered topology. The pheromone values are initially set by the soldier termites who identify the food source as well as the congenial settings. The pheromone value *τ* is considered to be proportional to the link utilization. Whenever packet flows through the edge (i,j), the pheromone value is updated for the path Pi,j by a factor *γ*, which is the pheromone deposit factor

*τ*_i,j_ = *τ*_i,j_ + *γ*

The value for *γ* is taken as a factor of the link capacity and when the pheromone value increases beyond a ceiling value, the path is assumed to be congested.

The pheromone is bound to evaporate and to account for the same, the pheromone value is multiplied by a decay factor, which is e-*δ*. That is

*τ*_i,j_ = *τ*_i,j_∗*e*^−*δ*^

Higher the rate of decay *δ*, the path can be assumed as congestion free.

The soldier termites secrete a different type of pheromone when there is an attack or on detection of a hostile environment. Analogous to the same,

*τ*_i,j_ = 0, When path is unavailable

*τ*_i,j_ = *pheromone*_*f*_*loor*, When path is available.

Like the soldier termites who constantly monitor the status such as soil temperature, moisture, food source density and hostile attacks, the SDN Controller shall be monitoring the link availability, congestion status and the link utilization. The priority of the traffic shall also be configured in the Controller. Once an available path without congestion is selected for a commodity, the flow table can be updated to set the path from source to sink. The pheromone deposit factor *γ* and the pheromone decay factor *δ* are the heuristic factors and are proportional to the bandwidth utilization of the selected path. Algorithm is given in Algorithm 1.

**Algorithm 1: Termite Inspired Optimization Algorithm**

***procedure*** Termite Inspired Optimization Algorithm

Input:

**P** ← Identified paths

c ← Link Capacity

k ← Commodity

p ← Priority of flow

*β* ← Congestion factor

*τ* ← Pheromone value

**Output**: Optimal path selection based on dynamic constraints

**Initialization**: Assign each path P_i_ with link capacity c_i_ and *τ*_icur_ as current pheromone-floor value based on availability

The Pheromone ceiling value for each path as *τ*_imax_ = *c*_i_ * *β*

1. Method:for each commodity k assumed as a termite colony
  1.1 Set Flow Groups F_k_ with priority P_k_ ← Worker Termites
2. for each higher priority F_k_
  2.1 Select high capacity available path P_i,j_ with (*τ*_icur_ <  = *τ*_imax_)
  2.2 Update Flow Table with the selected path for the FlowGroup
  2.3 Update *τ*_icur_ = *τ*_icur_ + *γ* /* Pheromone deposit factor
3. for each F_k_ of the commodity k
  3.1 If (selected path *τ*_icur_ >  =  pheromone_floor)
    3.1.1. If (*τ*_icur_ <  = *τ*_imax_) forward packet
    3.1.2. else *τ*_icur=_*τ*_icur_∗*e*^−*δ*^∕∗ pheromone decay for path
  3.2 else
    3.2.1. Select next available path for F_k_
4. Repeat above steps for all commodities k

***end procedure***

The Termite Inspired Algorithm mentioned in Algorithm 1 provides the movement of termite colonies and helps to avoid path congestion as well as path unavailability by identifying alternate paths dynamically. The termite swarm intelligence capability is modelled to identify alternate optimal paths in a dynamic manner.

### Experimental setup

**Figure 2:**
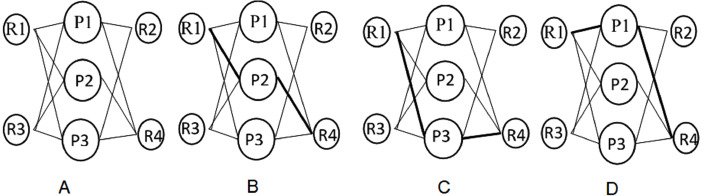
(A) Sample Topology; 2B: Shortest path from Site 1 to site 4; (C) Alternate shortest path from Site 1 to site 4; (D) Alternate Shortest path from Site 1 to site 4.

To evaluate the performance of the proposed Termite Inspired Algorithm, several tests under varying traffic demands were conducted. We selected a real network topology where four sites of an organization are connected through three Service providers as shown in Fig. 2A. The WAN links are assumed to be provided by different Service Providers (SP) P1, P2 and P3 and are of varying bandwidth. The edge routers R1, R2, R3 and R4 are connected to the multiple provider links to connect to the remote sites and are controlled by SDN Controller. As depicted in Figs. 2B, 2C and 2D, there are multiple shortest paths connecting site 1 and site 4. The objective is to achieve the maximum flow through the optimal path under dynamic traffic demands and constraints.

### Simulation

**Figure 3:**
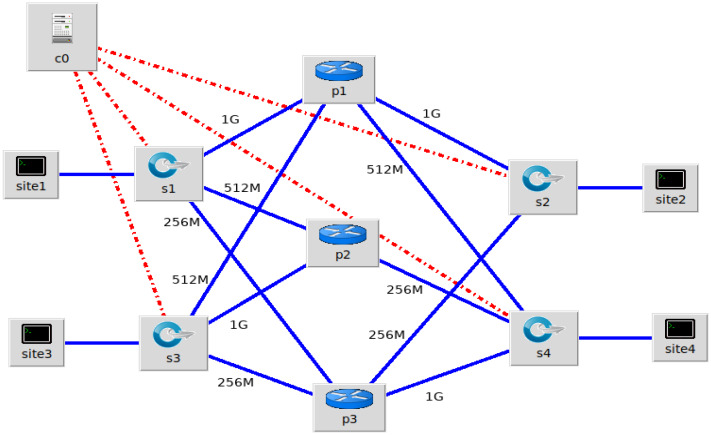
Mininet Network Topology for experiments connecting the four sites.

The simulation experiments were done on the Mininet (Mininet, 2018) platform which is the widely used simulator for SDN. The Mininet network topology chosen for the experiment is shown in Fig. 3. The topology consists of four Layer 3 OpenFlow enabled switches s1, s2, s3 and s4 as gateway routers, three routers P1, P2 and P3, to represent that of the WAN link Service Providers, one Controller C0 and hosts in each site. The Layer 3 switches were OpenFlow v 1.3 based and the SDN Controller was Floodlight v1.2 (Project Floodlight, 2018) The traffic generator used to generate traffic between the hosts is iperf (iPerf, 2018) which is the widely used traffic generator for creating TCP and UDP data streams. Iperf is used as a tool for active measurements of the maximum achievable bandwidth on IP networks.

From each site, the links were assumed to have bandwidth capacity of 1 Gbps, 512 Mbps and 256 Mbps respectively with the three different providers and the link weights were defined accordingly. The termite colony movements as UDP and TCP traffic with varying degree of priority were considered. A pheromone value of 70% of the link capacity is assumed as leading to link congestion. The Floodlight Controller was loaded with the proposed Termite Inspired Algorithm as part of the dynamic path allocation and link utilization management. We generated dynamic traffic flows of varying bandwidth demand iperf User Datagram Protocol (UDP) streams, iperf Transmission Control Protocol (TCP) streams. The traffic generated using **iperf** was programmed to range from 64 Mbps to beyond 1Gbps to test the limiting conditions and the behaviour of the algorithm. The pheromone deposit factor *γ* was assigned a value proportional to edge capacity and was of the range 10^−3^. The pheromone decay factor was assigned a random value between 0.01 and 0.07, the higher value being set for lower capacity edges.

The proposed algorithm was evaluated with respect to the Greedy Algorithm as presented by Awerbuch & Khandekar (2009). In the Greedy Algorithm, the flows will be looking at the current path’s network congestion and responds dynamically to changing congestion patterns. The simulations were performed under three scenarios, to represent typical enterprise network communications. TCP and UDP traffic with varying demands with equal priority and varying priorities were generated. Moreover, the links were made down during the traffic flow, to study the dynamic behaviour. Greedy algorithm could not handle the traffic with varying priorities as a factor. Both the proposed Termite Inspired Algorithm and the Greedy algorithm were executed and the results are discussed.

## Results and Discussions

### Scenario 1: All commodities with equal priority

**Figure 4:**
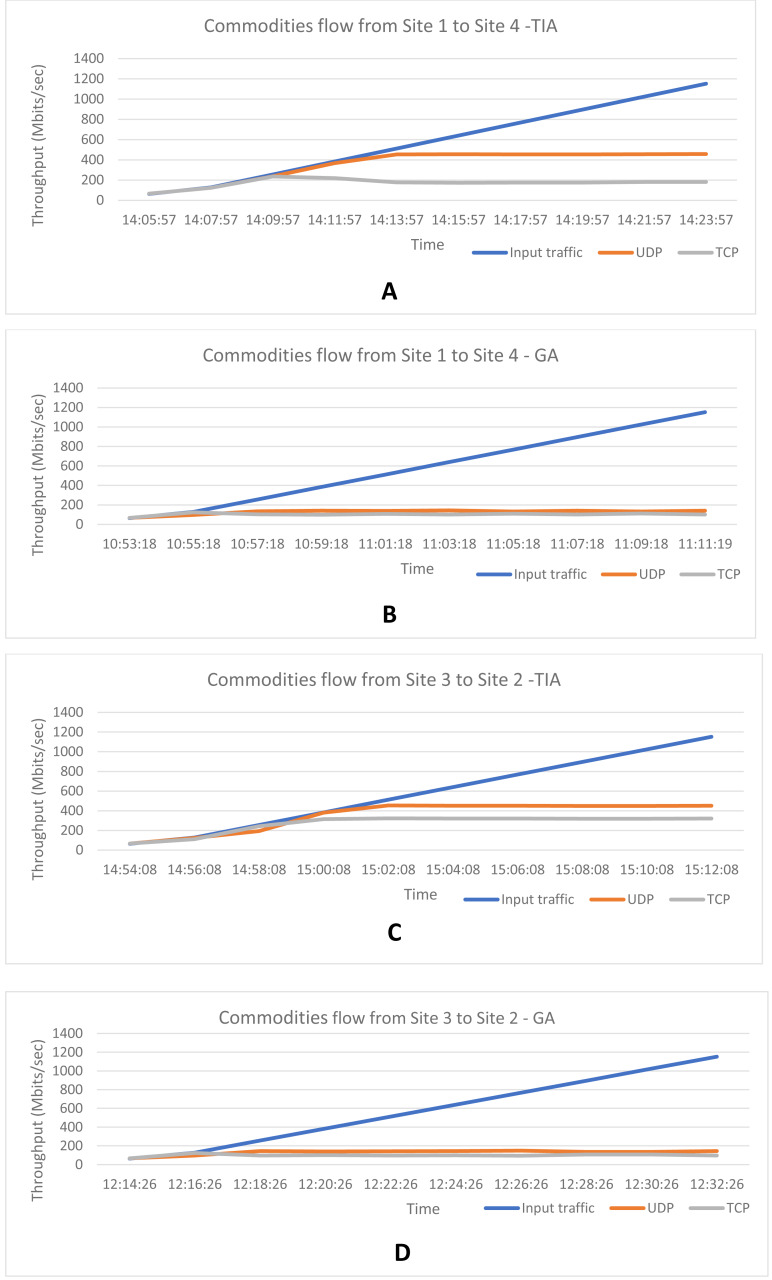
Throughput achieved based on dynamic UDP and TCP traffic inputs and the optimal path chosen for (A) TIA from site 1 to 4; (B) GA from site 1 to 4; C: TIA from site 3 to 2; (D) GA from site 3 to 2.

The iperf UDP and TCP traffic were generated simultaneously representing the multi commodity traffic. The traffic demands were increasing with time. Both the commodities were set with equal priority and the flow paths were identified. Tables 1 and 2 show the results of the proposed Termite Inspired Algorithm (TIA) for the commodities flowing from Site 1 to Site 4 and Site 3 to Site 2. It was seen that all the traffic flows were initially directed through the highest bandwidth path. When the total traffic demand was nearing congestion threshold, the traffic was automatically redirected to the next highest bandwidth path as highlighted in bold in Tables 1–4 show the result of the Greedy Algorithm (GA). It can be seen that the highest bandwidth path is chosen from each node in the case of the GA. The throughput depends on the capacity of the edges chosen for the path. The throughput achieved for both the UDP and TCP traffic based on the iperf generated input traffic and the edge capacities of the path chosen is depicted in the graphs shown in Figs. 4A–4D. From the graphs it is clear that the TIA achieves better throughput by choosing the optimal flow for the multiple commodities such as TCP and UDP traffic from site 1 to 4 as well as from site 3 to 2, when compared to GA.

**Table 1:** Flow Paths from Site 1 to Site 4, UDP and TCP traffic with equal priority traffic -TIA. All the traffic flows were initially directed through the highest bandwidth path p1. When the total traffic demand was nearing congestion threshold, the TCP traffic flow was automatically redirected to the next highest bandwidth path as highlighted in bold.

Commodities flow from Site 1 to Site 4							
UDP				TCP			
Input Traffic (Mbits/sec)	Path	Timestamp	Throughput (Mbits/sec)	Input Traffic (Mbits/sec)	Path	Timestamp	Throughput (Mbits/sec)
64	s1, p1, s4	14:05:57	67	64	s1,p1, s4	14:05:57	67.1
128	s1, p1, s4	14:07:57	124	128	s1,p1, s4	14:07:57	124
256	s1, p1, s4	14:09:57	237	256	s1,p1, s4	14:09:57	236
384	s1, p1, s4	14:11:57	368	384	**s1,p2, s4**	14:11:57	219
512	s1, p1, s4	14:13:57	455	512	**s1,p2, s4**	14:13:57	177
640	s1, p1, s4	14:15:57	456	640	**s1,p2, s4**	14:15:57	174
768	s1, p1, s4	14:17:57	454	768	**s1,p2, s4**	14:17:57	176
896	s1, p1, s4	14:19:57	454	896	**s1,p2, s4**	14:19:57	176
1024	s1, p1, s4	14:21:57	457	1024	**s1,p2, s4**	14:21:57	182
1152	s1, p1, s4	14:23:57	458	1152	**s1,p2, s4**	14:23:57	182

**Table 2:** Flow Paths from Site 3 to Site 2, UDP and TCP traffic with equal priority - TIA. All the traffic flows were initially directed through the highest bandwidth path from s3. When the total traffic demand was nearing congestion threshold, the TCP traffic flow was automatically redirected to the next highest bandwidth path as highlighted in bold.

Commodities flow from Site 3 to Site 2							
UDP				TCP			
Input Traffic (Mbits/sec)	Path	Time	Throughput (Mbits/sec)	Input Traffic (Mbits/sec)	Path	Time	Throughput (Mbits/sec)
64	s3, p2,s1,p1,s2	14:54:08	67.1	64	s3, p2,s1,p1,s2	14:54:08	67
128	s3, p2,s1,p1,s2	14:56:08	124	128	s3, p2,s1,p1,s2	14:56:08	111
256	s3, p2,s1,p1,s2	14:58:08	193	256	s3, p2,s1,p1,s2	14:58:08	242
384	s3, p2,s1,p1,s2	15:00:08	381	384	**s3, p1, s2**	15:00:08	316
512	s3, p2,s1,p1,s2	15:02:08	454	512	**s3, p1, s2**	15:02:08	323
640	s3, p2,s1,p1,s2	15:04:08	452	640	**s3, p1, s2**	15:04:08	321
768	s3, p2,s1,p1,s2	15:06:08	450	768	**s3, p1, s2**	15:06:08	322
896	s3, p2,s1,p1,s2	15:08:08	448	896	**s3, p1, s2**	15:08:08	320
1024	s3, p2,s1,p1,s2	15:10:08	449	1024	**s3, p1, s2**	15:10:08	319
1152	s3, p2,s1,p1,s2	15:12:08	451	1152	**s3, p1, s2**	15:12:08	322

**Table 3:** Flow Paths from Site 1 to Site 4, UDP and TCP traffic with equal priority traffic - GA.

Commodities flow from Site 1 to Site 4							
UDP				TCP			
Input Traffic (Mbits/sec)	Path	Time	Throughput (Mbits/sec)	Input Traffic (Mbits/sec)	Path	Time	Throughput (Mbits/sec)
64	s1, p1, s2, p3, s4	10:53:18	67.1	64	s1, p1, s2, p3, s4	10:53:19	67.1
128	s1, p1, s2, p3, s4	10:55:18	96.7	128	s1, p1, s2, p3, s4	10:55:19	124
256	s1, p1, s2, p3, s4	10:57:18	134	256	s1, p1, s2, p3, s4	10:57:19	104
384	s1, p1, s2, p3, s4	10:59:18	141	384	s1, p1, s2, p3, s4	10:59:19	99.7
512	s1, p1, s2, p3, s4	11:01:18	138	512	s1, p1, s2, p3, s4	11:01:19	107
640	s1, p1, s2, p3, s4	11:03:18	144	640	s1, p1, s2, p3, s4	11:03:19	101
768	s1, p1, s2, p3, s4	11:05:18	133	768	s1, p1, s2, p3, s4	11:05:19	110
896	s1, p1, s2, p3, s4	11:07:18	142	896	s1, p1, s2, p3, s4	11:07:19	101
1024	s1, p1, s2, p3, s4	11:09:18	132	1024	s1, p1, s2, p3, s4	11:09:19	111
1152	s1, p1, s2, p3, s4	11:11:19	141	1152	s1, p1, s2, p3, s4	11:11:19	101

**Table 4:** Flow Paths from Site 3 to Site 2, UDP and TCP traffic with equal priority traffic - GA.

Commodities flow from Site 3 to Site 2							
UDP				TCP			
Input Traffic (Mbits/sec)	Path	Time	Throughput (Mbits/sec)	Input Traffic (Mbits/sec)	Path	Time	Throughput (Mbits/sec)
64	s3, p2, s1, p1, s2	12:14:26	67.1	64	s3, p2, s1, p1, s2	12:14:26	67.1
128	s3, p2, s1, p1, s2	12:16:26	97.2	128	s3, p2, s1, p1, s2	12:16:26	124
256	s3, p2, s1, p1, s2	12:18:26	144	256	s3, p2, s1, p1, s2	12:18:26	97.8
384	s3, p2, s1, p1, s2	12:20:26	140	384	s3, p2, s1, p1, s2	12:20:26	101
512	s3, p2, s1, p1, s2	12:22:26	143	512	s3, p2, s1, p1, s2	12:22:27	97.8
640	s3, p2, s1, p1, s2	12:24:26	144	640	s3, p2, s1, p1, s2	12:24:27	98.8
768	s3, p2, s1, p1, s2	12:26:26	149	768	s3, p2, s1, p1, s2	12:26:27	94.3
896	s3, p2, s1, p1, s2	12:28:26	135	896	s3, p2, s1, p1, s2	12:28:27	107
1024	s3, p2, s1, p1, s2	12:30:26	135	1024	s3, p2, s1, p1, s2	12:30:27	107
1152	s3, p2, s1, p1, s2	12:32:26	144	1152	s3, p2, s1, p1, s2	12:32:27	97.6

### Scenario 2: Commodities with different priorities

The priorities of the commodities were set to high and medium. The flow paths were identified as shown in Tables 5 and 6 for the TIA. All the traffic flows were directed as per the algorithm. In scenario 2 also, the highest bandwidth path is chosen wherever possible without causing congestion. Greedy algorithm cannot accommodate the priority factor of commodities.

**Table 5:** Commodities with different priorities from site 1 to site 4- TIA.

UDP (Priority – High)				TCP (Priority –Low)			
Input Traffic (Mbits/sec)	Path	Time	Throughput (Mbits/sec)	Input Traffic (Mbits/sec)	Path	Time	Throughput (Mbits/sec)
64	s1,p1, s4	16:05:34	62	64	s1,p2, s4	16:05:35	61.7
128	s1,p1, s4	16:07:34	121	128	s1,p2, s4	16:07:35	120
256	s1,p1, s4	16:09:34	239	256	s1,p2, s4	16:09:35	238
384	s1,p1, s4	16:11:34	372	384	s1,p2, s4	16:11:35	221
512	s1,p1, s4	16:13:34	476	512	s1,p2, s4	16:13:35	209
640	s1,p1, s4	16:15:34	475	640	s1,p2, s4	16:15:35	197
768	s1,p1, s4	16:17:34	473	768	s1,p2, s4	16:17:35	192
896	s1,p1, s4	16:19:34	470	896	s1,p2, s4	16:19:35	192
1024	s1,p1, s4	16:21:34	468	1024	s1,p2, s4	16:21:35	189
1152	s1,p1, s4	16:23:34	471	1152	s1,p2, s4	16:23:35	191

**Table 6:** Commodities with different priorities from site 3 to site 2- TIA.

UDP(Priority – High)				TCP(Priority –Low)			
Input Traffic (Mbits/sec)	Path	Time	Throughput (Mbits/sec)	Input Traffic (Mbits/sec)	Path	Time	Throughput (Mbits/sec)
64	s3, p2, s1,p1,s2	15:32:25	56.8	64	s3, p1,s2	15:35:26	67.1
128	s3, p2, s1,p1,s2	15:34:25	111	128	s3, p1,s2	15:35:26	124
256	s3, p2, s1,p1,s2	15:36:25	194	256	s3, p1,s2	15:35:26	204
384	s3, p2, s1,p1,s2	15:38:25	302	384	s3, p1,s2	15:35:26	359
512	s3, p2, s1,p1,s2	15:40:25	329	512	s3, p1,s2	15:35:26	332
640	s3, p2, s1,p1,s2	15:42:25	351	640	s3, p1,s2	15:35:26	327
768	s3, p2, s1,p1,s2	15:44:25	354	768	s3, p1,s2	15:35:26	329
896	s3, p2, s1,p1,s2	15:46:25	352	896	s3, p1,s2	15:35:26	332
1024	s3, p2, s1,p1,s2	15:48:25	354	1024	s3, p1,s2	15:35:26	331
1152	s3, p2, s1,p1,s2	15:50:25	358	1152	s3, p1,s2	15:35:26	335

### Scenario 3: Commodity flow with link down conditions

The links in the commodity flow paths were made down. The TIA algorithm redirected the traffic flow through the available paths. The flow paths identified were as shown in Table 7. All the traffic flows were automatically redirected, as per the algorithm. In the case of GA, redirection did not happen as can be seen from Table 8.

**Table 7:** Commodity redirection on path down condition -TIA.

Links Unavailable at Timestamp	Input Traffic (Mbits/sec)	Path	Time	Throughput (Mbits/sec)
	64	s1,p1, s4	16:31:07	67.1
	128	s1,p1, s4	16:33:07	124
	256	s1,p1, s4	16:35:07	258
	384	s1,p1, s4	16:37:07	369
	512	s1,p1, s4	16:39:07	466
	640	s1,p1, s4	16:41:07	469
P1 down at 16:41:50	768	s1,p1, s4 & s1,p2,s4	16:43:07	310
	896	s1,p2,s4	16:45:07	244
P2 down at 16:45:28	1024	s1,p2,s4 & s1,p3,s4	16:47:07	232
	1152	s1,p3,s4	16:49:07	245

**Table 8:** No Commodity redirection on path down condition -GA.

Link down, Site1-Site4	UDP			
	Input Traffic (Mbits/sec)	Path	Time	Throughput (Mbits/sec)
	64	s1,p1, s2,p3,s4	11:45:55	67.1
	128	s1,p1, s2,p3,s4	11:47:55	134
	256	s1,p1, s2,p3,s4	11:49:55	243
	384	s1,p1, s2,p3,s4	11:51:55	244
	512	s1,p1, s2,p3,s4	11:53:55	246
	640	s1,p1, s2,p3,s4	11:55:55	246
P1 down at 11:59:15	768	s1,p1, s2,p3,s4	11:57:55	no datagram packet
	896		11:59:55	no datagram packet
	1024		12:01:55	no datagram packet
	1152		12:03:55	no datagram packet

The SDN Controller with the proposed TIA implemented has all the information required for computing the optimal paths and is capable for computing the routing paths of the gateway routers. If the gateway router is not SDN enabled, an OpenFlow enabled switch can be introduced prior to the gateway router, so that all the traffic flowing through the gateway router is controlled traffic. Extensive tests were carried out to validate the correctness and performance of the algorithm. In the sample mesh topology connecting four distant sites, traffic management with dynamic path selection was studied, which was influenced by network performance characteristics such as bandwidth, congestion and network traffic prioritization. In each scenario, the capacities of the links were fixed and the traffic was introduced dynamically to populate links with sufficient activity as to represent typical enterprise network communications. The results indicate that the proposed algorithm provides better throughputs, redirects through less congested paths dynamically. The algorithm can handle additional constraints such as priority. Link failures are common in real world networks. The algorithm also handles the link down condition by automatically redirecting the traffic through the available paths. The traffic patterns were generated randomly for testing the robustness of the algorithm. Simulation results show the allocation of optimal paths with dynamic traffic conditions. The algorithm supported end to end path decisions based on the conditions that exist on the links. It was observed that the high priority traffic always chose the high bandwidth path. When congestion is detected, the next available path for the flow group is chosen dynamically. The proposed algorithm considers the link capacity, congestion factor, link availability and priority dynamically in selecting the optimal end to end path.

### Comparison with other popular Swam based algorithms

The default algorithm present in the Floodlight SDN controller is the Dijkstra’s algorithm which computes the available shortest path. Since the existing modules in SDN Controller are unable to achieve the object function with constraints, initially, the popular swarm based algorithms for routing namely, the Ant Colony Optimization (ACO) (Di Caro, 2004), Bee Colony Optimization (BCO) (Saritha & Vinod Chandra, 2019) and Particle Swarm Optimisation (PSO) (Yao et al., 2016) were considered.

In the current problem, the multiple paths to the destination are known and the optimal path for maximal flow based on multiple dynamic constraints has to be identified on a continual basis. Pheromone intensity is considered as an indication of congestion factor. As the pheromone intensity increases beyond the threshold value, an alternate path for the commodity is to be identified. In the existing ACO, the concept of guard ant signalling an alert like soldier termite is not implemented. Hence for continual optimisation based on link availability, ACO will track the pheromone intensity and remove the path once the intensity falls below a threshold.

BCO algorithm was selected for comparison because the honey bee swarms also exhibit behaviour like termites and have similar division of labour with employee, onlooker and scout bees. Each food source represents a candidate solution and the nectar amount associated represents the quality of food. The quality of food was associated with congestion factor. The PSO algorithm considered the flow demand, the link capacity and the forwarding table size.

All the algorithms identified the same optimal paths for the commodities to begin with. But they failed for a new path identification on link failure. Similarly, the three algorithms did not have models supporting for prioritised traffic. Selecting a new path as part of congestion avoidance suffered a latency in the case of BCO and PSO as part of the communication pattern and fitness evaluation. For the ACO algorithm, the probabilistic selection of the next available path instead of a congested path introduced latency. In the case of termite inspired algorithm, the clear division of labour and multiple pheromone trails are added advantages in modelling the system.

### Physical test bed evaluation

To evaluate the algorithm in a real scenario, a physical test bed was set up in the lab. The topology was realized using commodity hardware. The network consists of traditional network elements in the form of edge routers. The SDN elements such as a Controller and data plane switches were introduced transforming the setup into a hybrid network. The testbed consists of a server platform for the SDN Controller C0, OpenFlow enabled switches S1, S2, S3 and S4 and four edge routers R1, R2, R3 and R4 and some terminals. Specifically, Floodlight v1.2 executing on Ubuntu v16.04 was chosen as the SDN Controller software. The Controller executed in an Intel Xeon platform with 64 GB RAM. The SDN data plane switch which interfaced with the traditional edge router was implemented using Cisco Catalyst 9300 Series switch, which supported the OpenFlow protocol. We used laptops as the terminals which generated the required flows for testing the algorithm loaded into the Controller. The edge routers used were Juniper SRX220 Routers with support for 8 ports. The connecting links were configured for varying bandwidths of 1 Gbps, 100 Mbps and 10 Mbps. The termite inspired optimization algorithm for dynamic multipath routing and congestion avoidance was loaded in the Floodlight Controller. The policies were formulated in such a way that UDP traffic was given high priority and TCP traffic given medium priority. The upper threshold for the pheromone, i.e., the pheromone ceiling value was set as 70% of the link capacity for each link, exceeding which alternate paths for the flow group of the commodity were to be chosen dynamically. Routers were configured to forward the flows from the data plane switch ports which were controlled by the SDN Controller.

The algorithm performed similar to the Mininet simulation. The high priority UDP traffic was directed to the higher bandwidth path of 1 Gbps and the lower priority TCP traffic was directed to the lower bandwidth 100 Mbps path. The throughput reached the maximum configured flow avoiding congestion. Analogous to the termite behavioural adaptation to the environment with their organizational and anatomical support to carry out functions, the proposed setup also adapts dynamically to the changing traffic demands, link conditions and traffic priorities. The key elements include the SDN Controller which monitors like the soldier termites, the polices which are like the behaviour pattern of the swarm, the forwarding device which forwards the packets similar to the role of the worker termites and the Controller analytics with the implemented algorithm (which react to the changing network conditions such as link availability and link load additions), similar to the termite colony pheromone strategies.

In legacy WAN, traffic is considered in terms of packets, whereas in the case of SDN, traffic is considered in terms of flows. The practical factors and the existing constraint in replacing the legacy routers made us design a hybrid solution by introducing SDN enabled switches at the gateway of the network. The proposed setup facilitated the control of a set of flows with the maximum traffic amount and without affecting the performance in the case of dynamic traffic requirements.

## Conclusions

In the existing networks, static end-to end resource allocation is used to meet the WAN traffic demands. This often leads to resource overprovisioning and lacks knowledge of the current status and demands. The proposed algorithm provided the required traffic engineering with dynamic multipath routing with dynamic constraints. SDN provides a better way of network orchestration for traffic management. Apart from dynamic rerouting, another major requirement is bandwidth provisioning. Static bandwidth provisioning is inefficient considering the varying demands from applications. The proposed bioinspired algorithm based on Termite behavior is a novel algorithm that can be implemented in the SDN Controller to support elastic bandwidth demands from applications, by avoiding congestion and based on link availability.

Our future works include implementing the algorithm in higher bandwidth backbones as well as in frugal bandwidth scenarios for the effective utilization of the available bandwidth under dynamic conditions. The operational impact of latency and jitter are to be considered within the scope of the algorithm.

##  Supplemental Information

10.7717/peerj-cs.283/supp-1 Supplemental Information 1 Java source code for the Termite Inspired Algorithm embedded in the Floodlight Controller

## Additional Information and Declarations

### Data Availability

The following information was supplied regarding data availability: The Java source code corresponding to the proposed algorithm is available in a Supplementary File.
